# Psychological Intervention Based on Mental Relaxation to Manage Stress in Female Junior Elite Soccer Team: Improvement in Cardiac Autonomic Control, Perception of Stress and Overall Health

**DOI:** 10.3390/ijerph20020942

**Published:** 2023-01-04

**Authors:** Eleonora Pagani, Naomi Gavazzoni, Giuseppina Bernardelli, Mara Malacarne, Nadia Solaro, Emanuele Giusti, Gianluca Castelnuovo, Piero Volpi, Giulia Carimati, Daniela Lucini

**Affiliations:** 1 Department of General Psychology, Catholic University, 20123 Milan, Italy; 2 DISCCO Department, University of Milan, 20122 Milan, Italy; 3 Exercise Medicine Unit, Istituto Auxologico Italiano, IRCCS, 20135 Milan, Italy; 4 BIOMETRA Department, University of Milan, 20129 Milan, Italy; 5 Department of Statistics and Quantitative Methods, University of Milano-Bicocca, 20126 Milan, Italy; 6 Psychology Research Laboratory, Istituto Auxologico Italiano IRCCS, 20149 Milan, Italy; 7 Department of Psychology, Catholic University, 20123 Milan, Italy; 8 Clinical Psychology Research laboratory, Istituto Auxologico Italiano, IRCCS, 28824 Verbania, Italy; 9 IRCCS Humanitas Research Hospital, 20089 Milan, Italy

**Keywords:** stress management, autonomic nervous system, sport performance, mental training, soccer, psychology, HRV

## Abstract

Chronic stress may represent one of the most important factors that negatively affects the health and performance of athletes. Finding a way to introduce psychological strategies to manage stress in everyday training routines is challenging, particularly in junior teams. We also must consider that a stress management intervention should be regarded as “efficacious” only if its application results in improvement of the complex underlying pathogenetic substratum, which considers mechanistically interrelated factors, such as immunological, endocrine and autonomic controls further to psychological functioning and behavior. In this study, we investigated the feasibility of implementing, in a standard training routine of the junior team of the Italian major soccer league, a stress management program based on mental relaxation training (MRT). We evaluated its effects on stress perception and cardiac autonomic regulation as assessed by means of ANSI, a single composite percentile-ranked proxy of autonomic balance, which is free of gender and age bias, economical, and simple to apply in a clinical setting. We observed that the simple employed MRT intervention was feasible in a female junior soccer team and was associated with a reduced perception of stress, an improved perception of overall health, and a betterment of cardiac autonomic control. This data may corroborate the scientific literature that indicates psychological intervention based on MRT as an efficacious strategy to improve performance, managing negative stress effects on cardiac autonomic control.

## 1. Introduction

Stress is one of the most used or abused words in the world, at least in the western part. There are many meanings attached to the concept of stress, reflecting its psychological, physiological, behavioral, or social aspects [1,2,3]. The definition to which we will refer [4], in the full awareness that it might be improved, is: “stress may be considered as the psychological, behavioral and physiological (or pathophysiological) consequence of the interaction between a subject and a stressor; considering as “stressor” everything (acute or chronic) present in the environment or in the subject’s mind that could be perceived as important, dangerous or potentially capable to modify, both negatively or positively, the subject’s life”. Stress per se [5] is a physiological, useful response [2] to re-establish homeostasis through a complex process. This implies regulatory systems (hormonal hypothalamic-pituitary-adrenocortical chain, autonomic nervous system, and immunity) [4,5,6,7,8,9] modulated by the subjective perception of stress, individual (genetic, biological, psychological) differences and behavior [9]. The unfavorable nature of stress becomes manifest when the final result is not adaptation and homeostasis, but when bodily symptoms or illnesses, such as coronary artery disease and functional syndromes [3,8,10,11,12] or psychosocial effects (deterioration of mood, unwillingness to change, isolation, etc.) appear. The attention to stress in sports is growing progressively. While acute stress may be a normal, useful adaptive response to a stimulus (even if it can affect performance [13]), chronic stress may represent one of the most important (often the main) factors that may negatively affect the health and performance of athletes [14,15,16,17]. Stress management is nowadays considered a fundamental strategy to improve athlete’s achievements and to reduce the occurrence of sport injuries; consequently, multiple sport psychology techniques are available, at least in elite settings. The results of a recent meta-analysis [18] were supportive of using sport psychology procedures to help improve sport performance, confirmed that variations in psychological constructs relate to variations in results, and showed that mindfulness interventions were associated with higher beneficial effects on performance [19,20,21]. On the other hand, finding ways to introduce psychological strategies to manage stress in everyday training routines is challenging, particularly for young athletes. Multiple barriers may be considered, for instance, time and cost constraints, athletes’ and team managers’ predisposition towards psychological interventions in the face of busy training programs. It is also important to recall that a stress management intervention should be considered “efficacious” only if its application results in improvement of the complex underlying pathogenetic substratum, recalling that immunological, endocrine, and autonomic controls further to psychological functioning and behavior, are all mechanistically interrelated [6,22]. From a practical point of view, the link between the autonomic nervous system (ANS) and stress is well studied, and a large body of literature is available to demonstrate a clear cardiac autonomic (CAR) impairment in chronic stress suggesting a significant pathogenetic role in many chronic situations [4,7,8,9,22,23,24,25,26,27], ranging from ischemic heart disease to metabolic syndrome, and acute conditions, such as sudden death [28]. This latter event is particularly relevant in sports [29]. The importance of ANS in this context is also corroborated by studies that clearly show a lessening of the autonomic impairment associated with stressful conditions following stress management programs [4,26,30]. These programs also considered techniques that involve regularization of breathing patterns, such as mental training, mindfulness, yoga, and even rosary prayer [31,32,33,34,35]. The importance of cardiac autonomic control is moreover emphasized by its role in determining cardiovascular control and adaptation to exercise [36]. Cardiac ANS regulation and responsiveness are presently considered an important element in determining athletes’ performance, including other factors, such as cardiorespiratory fitness, body composition, and psychology [37,38]. Within these multidimensional determinants of athletes’ performance, the autonomic nervous system plays a special role [37] as a bridge between physical and psychological elements. Spectral analysis of Heart Rate Variability (HRV) represents today the de facto standard method to assess the features of autonomic cardiac regulation in clinical and, particularly, in sport settings [36,37,38,39], being also simple, economical, and non-invasive. HRV appears particularly useful in sports as a means to follow various training steps [40], also using miniaturized portable instruments [41]. Nevertheless, the complexity of analytical techniques and difficulty of interpretation, in particular, if mechanistically considering one autonomic index at a time, close to the well-known influence of age and gender on CAR, may act as a barrier to more extensive use of HRV in practice, particularly during dynamic conditions, such as exercise [42,43]. Given the fundamentally unitary nature of visceral neural regulation [44], particularly in the case of exercise-related cardiac performance, in order to overcome some of these barriers, we introduced an integrated, unitary Autonomic Nervous System Index (ANSI), which represents a single composite percentile-ranked proxy of autonomic balance, whereby higher values indicate better autonomic control. ANSI is extracted from the far more complex autoregressive spectral analysis of HRV by combining the three most informative indexes (i.e., RR interval, RR variance, and stand–rest difference in RR low-frequency component expressed in normalized units) [45,46,47]. ANSI is, by design, free of age and gender bias and correlates with cardiac baroreflex sensitivity, which is considered an important cardiac prognostic predictor [48]. In a previous paper [49], we observed that this index was higher in national A series soccer players compared to age-matched controls, being highest in midfielders and defenders. ANSI was capable of differentiating different sports training [50] and improved after endurance training in elite basket players [51]. A more complex version of this index (which also considers autonomic parameters related to recovery after exercise stress test) shows that a better CAR differentiated Italian athletes who were eventually selected for participation in the 2016 Rio Olympic Games from those who were not [38].

The aim of the present study was to investigate the feasibility of implementing, in a standard training routine of the junior team of the Italian major soccer league, a stress management program based on mental relaxation training (MRT) and evaluate its effects on cardiac autonomic regulation and stress perception.

## 2. Materials and Methods

**Figure 1:**
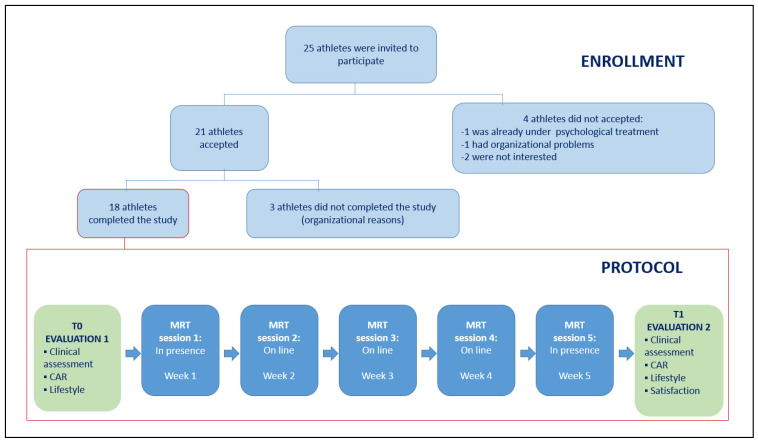
Flow chart of the enrollment procedure and protocol.

All twenty-five female athletes (aged 16.06 ± 0.6 years) of a junior team of the Italian major soccer league (“Serie A”) were offered to participate in the study (see Figure 1).

A group of twenty-one athletes (aged 16.06 ± 0.6 years) accepted and were enrolled in this study. Good health was ensured in athletes by their team doctor (following Italian law that prescribes annual preparticipation screening in competing athletes). Participants were first evaluated when enrolled (T0) and after five weeks (T1), during which they followed a mixed (on-site and web-based) mental relaxation training program (MRT) based on both Schultz Autogenous Training [52,53,54] and mindfulness [55,56,57], both adapted to a sport setting. Eighteen athletes completed the study, while three dropped out due to organizational reasons.

The evaluations considered are shown in the following sections.

### 2.1. Clinical Assessment

Anthropometric (Height, Weight, Waist Circumference) and Hemodynamics Parameters (Systolic/Diastolic Arterial Pressure and Resting Heart Rate) were assessed.

### 2.2. Cardiac Autonomic Regulation (CAR)

Recordings were performed between 2:00 pm and 5:00 pm in an ad hoc equipped room located near the locker room of the football training field. After a preliminary nominal 10 min rest period in the supine position, ECG and respiratory activity (piezoelectric belt) were continuously recorded over a minimum 5 min period with a two-way radiotelemetry system (Marazza, Monza, Italy). Subsequently, subjects were asked to stand up unaided and remain in the upright position for 5 min, during which recordings were maintained. Data were acquired with a PC at 250 samples/second using a custom-built software tool (HeartScope) that automatically provided a series of indices describing HRV in the time domain: RR interval (in msec) and RR interval variability (assessed as total power, i.e., variance, in msec^2^), taken as simple classifiers typical of vagal control [47,58,59]; and in the frequency domain: autoregressive spectral components both in the low frequency (LF, center frequency ≈ 0.1 Hz) and in the high frequency (HF, centered with respiration, ≈0.25 Hz), assessed in msec^2^ as well as in normalized units (nu). To include an approximate evaluation of the effects of sympathetic activation produced by active standing, the stand-rest difference (Δ) in LFnu was also computed. Arterial pressure was assessed using an electronic sphygmomanometer. Recently to simplify the clinical interpretation of multiple HRV variables, we proposed a unitary autonomic index (ANSI) [47] regarded as a proxy of cardiac autonomic regulation. ANSI was derived as a combination of factor analysis results and a clinically optimized radar plot. By applying factor analysis to a multitude of HRV indices, RR, RR interval variance, and ΔRR LFnu were found to be highly representative of the cardiac autonomic information (considering amplitude and oscillatory code modalities) [45]. Accordingly, ANSI was constructed following this procedure: first, the percentile rank (PR) transformations of RR, RR interval variance, and ΔRR LFnu are computed within each age-by-gender class. This way, new variables adjusted for age and gender effects are obtained; second, for each subject, a radar plot is built with the values of these three PR transformed variables and the area of the thus obtained triangle computed; third, the PR transformation is applied to the triangle areas to obtain ANSI as a composite normalized indicator ranging in [0, 100] [60].

### 2.3. Lifestyle Assessment

An ad hoc questionnaire to quantify lifestyle [61,62,63] was employed:

Physical activity (total activity volume) was assessed by a modified version of the commonly employed short version of the International Physical Activity Questionnaire (IPAQ) [63], which focuses on intensity (nominally estimated in Metabolic Equivalents-MET- according to the type of activity) and duration (in minutes) of physical activity. We considered the following levels: brisk walking (≈3.3 METs), other activities of moderate intensity (≈4.0 METs), and activities of vigorous intensity (≈8.0 METs) [62].

Nutrition was assessed using the American Heart Association Healthy Diet Score [62], taking into consideration fruit/vegetables, fish, sweetened beverages, whole grain, and sodium consumption (the assessment of this latter factor was adapted to Italian eating habits).

Sleep was assessed by simply inquiring about the number of hours on average slept every night and asking about the perception of sleep quality (see below).

Stress and somatic symptoms perception were assessed using a self-administered questionnaire [26,61], providing nominal self-rated scales (higher values indicate higher degrees of symptoms) that focused on: (i) the appraisal of the overall stress and fatigue perception by evaluation scales with integer scores from 0 (‘no perception’) to 10 (‘highest perception’) for each measure; (ii) the Short-Subjective Stress-related Somatic Symptoms Questionnaire (4S-Q), inquiring about four somatic symptoms accounting for the majority of somatic complaints. For scoring purposes, each response was coded from 0 (‘no feeling’) to 10 (‘a strong feeling’); thus, the total score ranged from 0 to 40. Moreover, perceptions of quality of life, sleep, and overall health were assessed using evaluation scales from 0 (‘worst quality’) to 10 (‘best quality’) for each measure.

### 2.4. Mental Relaxation Training (MRT)

An MRT protocol was designed considering time, organizational and economic barriers which frequently may hamper its realization in elite junior soccer teams. It consisted of five weeks of intervention, one session (60 min) every week. The training was delivered by a certified psychologist. The first and last sessions were held at the facility of the soccer training field in order to avoid transportation time and minimize cost and organizational issues while maintaining the positive interpersonal relationship of athletes with the psychologist and with all groups. The other sessions were held online using Microsoft Teams in order to further reduce organizational and time constraints. The training goals were to improve resources to manage stressful conditions linked to competition and reduce physiological/physio-pathological arousal. The protocol considered both Schultz Autogenous Training [52,53,54] and mindfulness [55,56,57], both adapted to a sport setting. Athletes were invited to focus their attention on the present time, their individual respiratory patterns, and bodily sensations.

At the end of the last training session, we asked athletes to fill out a short questionnaire in order to evaluate their thought about the potential value of the training; in particular, we considered important the answer (choosing between “Yes” or “No”) to the following questions: “Do you consider the training useful?”, “Would you like to continue the training by participating in other sessions?”.

All subjects and their parents had provided informed consent at the time of the visit; they were informed and agreed that their anonymized data could be used for statistical or scientific projects. The protocol of this study followed the principles of the Declaration of Helsinki and Title 45, US Code of Federal Regulations, Part 46, Protection of Human Subjects, Revised 13 November 2001, effective 13 December 2001, and was approved by the Ethics Committee of University of Milan (report dated 14 December 2021). All subjects and their parents gave their written informed consent.

### 2.5. Statistical Analysis

Data are presented as median ± MAD (Median Absolute Deviation) computed for the 21 considered variables measured in rest at times T0 and T1 (Figure 1). The same summary statistics were calculated for the “stand-rest” differences (“∆ variables”) of the HRV indices evaluated at both T0 and T1. Significant differences between variables at T0 and T1 were assessed using two non-parametric tests for paired data, the sign test and the Wilcoxon signed-rank (WSR) test [64]. Significant results obtained on both tests provided stronger empirical evidence of the presence of intervention effects. *p*-values were computed based on the exact test statistic distributions (i.e., they were not asymptotic). The nominal significance level was set to 0.05.

The small number of subjects (18 in all) requires some caution. Although it is not a barrier in itself [65], it calls for careful interpretation, avoiding generalizations, and preferring inferential methods, such as the exact distribution-free tests we considered, that do not require normally distributed variables and are relatively not affected by small sample sizes. All the statistical analyses were performed with a commercial package (IBM SPSS 28, Armonk, NY, USA).

## 3. Results

Adherence to training: on average, 89% of athletes were present at every training session and no one of them was absent more than one time. All athletes considered the training useful; 11 (62.2%) subjects declared their desire to receive more training sessions, while seven (38.8%) subjects did not. Table 1 reports the analysis results of the subjects’ anthropometric/hemodynamics parameters and lifestyle assessment data at T0 and T1. Both sign and WSR tests proved that Systolic Arterial Pressure differed significantly in comparing T0 and T1 (Sign test: *p* = 0.031, WSR test: *p* = 0.004), while diastolic arterial pressure (*p* = 0.028) and heart rate (*p* = 0.031) achieved a significant result only on the WSR test. Overall, median values of SAP, DAP, and HR appear to be slightly reduced after MRT. As expected in athletes, total activity volume was elevated both before and after MRT; the American Heart Association Healthy Diet Score was slightly greater after MRT. However, no significant result was observed in such cases. Regarding the perception and symptom scales, an apparent reduction in the median scores of perception of stress, 4SQ, and perception of tiredness was observed after MRT, while the median scores of perception of sleep quality and perception of overall health slightly increased after MRT. No difference was noted instead regarding the perception of quality of life and slept hours. However, significant differences resulted only in the perception of stress (sign test: *p* = 0.035, WSR test: *p* = 0.015) and perception of overall health (sign test: *p* = 0.021, WSR test: *p* = 0.021).

Table 2 reports the results of relevant variables derived from the analysis of HRV. No significant changes were noted regarding all the HRV variables comparing the rest condition at T0 with the rest condition at T1. Vice versa, considering the changes induced by active orthostatism (standing—the simplest physiological stimulus capable of inducing sympathetic activation), significant differences after MRT were observed for HR (ΔHR mean) (both sign and WSR tests: *p* < 0.001); the marker of sympathetic activation (ΔRR LFnu) (sign test: *p* = 0.031, WSR test: 0.014); the ratio of LF and HF (ΔRR LF/HF) (WSR test: *p* = 0.005). Importantly, we noticed an increased response after MRT in all these cases. That suggests a greater capacity of the autonomic nervous system to react to standing. Moreover, ANSI, the composite index proxy of CAR, differed significantly comparing T0 and T1 (WSR test: *p* = 0.030) and was clearly improved (from 35.87 ± 13.99 to 57.55 ± 28.88), thus indicating a betterment of CAR after MRT.

## 4. Discussion

In this study, we showed that MRT was associated with a reduction in arterial pressure, an improvement of cardiac autonomic control, a reduction in stress perception, and a betterment of overall health perception in female junior elite soccer players. Moreover, this study evidenced the feasibility of implementing an MRT in a real training routine of junior elite athletes. Cardiac autonomic control represents one of the main mechanisms impaired by stressful events. ANS imbalance following acute stress (such as the one characterizing sports competitions), in combination with maximal exercise intensity and genetics, may be responsible for major ominous events such as sudden cardiac death [28,29]. The increased risk and worst prognosis of cardiovascular and metabolic diseases linked to chronic stress may also be due to a direct action on CAR [3,7,8,9]. Moreover, chronic stress may indirectly undermine CAR favoring unhealthy lifestyles (such as changes in training routine in athletes or sedentariness in non-athletes, unhealthy nutrition, smoking, worse sleep hygiene, less adherence to medication, etc.), which per se may negatively affect autonomic nervous system control [4]. The study of CAR in subjects experiencing stress conditions may represent a challenging issue, considering that the use of any invasive technique or technique perceived as dangerous may per se be a stressor. On the other hand, simply considering variables that are under ANS control (such as heart rate, arterial pressure, skin conduction, etc.) but not representing a “measure” of ANS may be considered misleading and not sufficient to investigate ANS accurately. The study of heart rate variability (HRV) nowadays represents the goal standard, a non-invasive, non-intrusive technique to study ANS in clinical settings. The possibility today to overcome many technical and interpretative limitations (such as the employed algorithms to analyze the recorded electrocardiographic signal, the used protocol, the large set of potentially redundant variables obtained from the analysis, the influence of gender and age bias, etc.) [38,47] reinforces HRV as the “de facto” technique to assess ANS in clinical settings, particularly when the goal is to evidence the effects of stressful conditions [66]. More than 22,000 results in the Medline database respond to the item heart rate variability or HRV; of these, 1678 respond to the combination with mental or psychological stress, demonstrating a strong interest from the scientific community in the use of HRV to study stress. Our group also contributed to this issue. In previous papers, we showed an impairment of CAR both in acute and chronic [4,24,26] stress conditions, a clear relationship among perception of stress, poor lifestyle, and CAR impairment [67], and its improvement with several interventions [25,26,30,67] strategies. Of particular interest is the observation that stress management programs, particularly those based on mental relaxation, are capable of improving CAR in addition to reducing stress/tiredness perceptions and stress-related somatic symptoms perception [25,26]. Several mental relaxation techniques are based on the regularization of breathing, considering slow, regular respiration where the expiratory phase is longer than the inspiratory one. This voluntary regular respiratory pattern is likely to activate autonomic afferent pathways, which determine a feed-backed vagal efferent response, with consequent affects at the periphery characterized by reduced heart rate, reduced arterial pressure, reduced muscular tension, etc., underlying the structural and functional link between somatomotor, respiratory control mechanisms with ANS. The regular use of these techniques may also result in an improvement of immunological and endocrine control, positive results that may also be explained considering the important link between those control mechanisms with ANS [33]. Studies employing HRV showed a reduction in sympathetic control and an improvement in vagal control [25,26,31,34,35] both in healthy subjects and in patients. In this paper, we described an improvement in CAR as evidenced by the increase in ANSI. In particular, we observed an increased capability of the system to counterbalance the active orthostatic stimulus following MRT. This observation may be of clinical importance because it allows for verifying the effectiveness of a planned intervention to manage stress in a real sports setting. In fact, an intervention may be considered efficacious only if its application results in evidence of improvements in the underlying pathogenetic mechanisms. On the other hand, other mechanisms may be improved by MRT, in particular, psychological functioning, perception of stress, self-efficacy, coping strategies, self-esteem, etc. In this paper, we do not address these important aspects in depth. For time and budget constraints, we limited our observation to the simple perception of stress, tiredness, stress related somatic symptoms, quality of life and quality of health, using an ad hoc questionnaire that we already employed in several studies [26,61,68]. This questionnaire was, for instance, capable of unveiling a relationship between higher stress perception and worse performance in elite rowers [69] and in Olympic athletes competing in skill-based disciplines [38]; of showing a reduction in stress perception in employees following a stress management program [26], in metabolic syndrome or oncologic patients following a lifestyle management program [67], in bariatric patients following prehabilitation and subsequently surgery [70]. In the present study, we observe a reduction in the perception of stress and an improvement in the perception of overall health. Importantly, no changes were observed in the total physical activity volume, suggesting that the observed changes were not linked to a different training load. While the importance of psychological programs to improve athletes’ performance and well-being is nowadays appreciated by elite professionals and their teams, which offer ad hoc programs to the athletes, such programs are less offered in junior teams. There are multiple potential barriers to consider, and several of them are related to time, economic, and organizational constraints. We designed the intervention employed in this study, considering these barriers, proposing a simple, short intervention (only five encounters), considering a minimum (the first and the last ones) of face-to-face sessions and delivering the other sessions using the web in order to minimize organizational issues and dedicated time, but preserving (at least in part) the important value of the interpersonal relationship. Moreover, the assessment of physiological and psychological parameters was simple, requiring little time (15 min for both ANS evaluation and questionnaire filling), carried out at the soccer training field, minimizing transportation time and saving time for training and personal school homework. This protocol encountered the favor of both young athletes and their families and showed the feasibility of implementing an MRT in a real training routine of junior elite athletes. All athletes considered the training useful; 11 (61.1%) of them declared their desire to receive more training sessions, while seven (38.9%) did not. Interestingly, the improvement of ANSI after MRT was greater and significant (according to the one-tailed sign and WSR tests) in the subgroup who declared the desire to receive more MRT sessions (median ± MAD before: 40.70 ± 10.07; after: 70.77 ± 23.08; ∆ANSI: 21.82 ± 19.16; one-tailed sign test: *p* = 0.033; one-tailed WSR test: *p* = 0.016) as compared to the subgroup who did not (median ± MAD before: 28.25 ± 12.31; after: 49.37 ± 23.92; ∆ANSI: −0.84 ± 6.85; one-tailed sign test: *p* = 0.500; one-tailed WSR test: *p* = 0.406). However, given the small subgroup sizes, we regarded these results as useful indications only for further studies and did not proceed to more in-depth investigations in the present context.

The observation that stress may facilitate the occurrence of sport injuries and that stress management programs may be a valuable strategy to reduce them are of paramount importance in the field of athletics [71]. In this context, the possibility to apply and verify the effects of a simple and sustainable stress management protocol may be useful.

Limitations: this study presents some limitations: firstly, the group size was small, limiting the interpretative value of results; we have to consider that the number of female athletes of the major league (“Serie A”) junior team was limited to twenty-five, and we enrolled twenty-one of them. Secondly, the protocol that we proposed was very short (only five sessions, one each week) in order to actually obtain results over a short time. Then, we did not consider a control group: we would like to enroll another junior team of same gender and age in the study as the control group, but this time it was not possible. On the other hand, we observed in other studies that sham interventions did not modify CAR per se [25,26], supporting the idea that the changes were attributable to functional remodeling. Another important limitation is that we assessed only CAR and individual perception of stress and quality of health and quality of life; we did not consider other known control mechanisms potentially involved in explaining the benefit related to MRT, particularly hormonal changes that occur with stress.

## 5. Conclusions

In this study, we observed that a simple intervention based on mental relaxation training was feasible in a female junior soccer team. This intervention was associated with a reduced perception of stress, improved perception of overall health, and betterment of cardiac autonomic control. These are all important mechanisms implied in the pathogenesis of the negative effects of acute and chronic stress. This data, even preliminary and related to a small size of elite athletes, may corroborate the scientific literature that indicates psychological intervention based on mental relaxation as an important strategy to improve performance, managing negative stress effects on cardiac autonomic control, which may be assessed employing HRV. Further studies on a larger cohort of athletes, also considering a control group are needed to corroborate the present findings possible taking into consideration also the assessment of other important control mechanisms such as immune and hormonal controls.

## Figures and Tables

**Table 1:** Subjects’ anthropometric/hemodynamics parameters and lifestyle assessment data at T0 and T1: summary statistics (median ± MAD) and two-tailed non-parametric statistical tests.

Variables	Median ± MAD (Time 0)	Median ± MAD (Time 1)	Sign Test Significance	WSR Test Significance
SAP [mmHg]	110.00 ± 7.00	102.00 ± 7.00	**0.031**	**0.004**
DAP [mmHg]	58.00 ± 4.00	54.50 ± 4.50	0.077	**0.028**
HR [b/min]	61.50 ± 8.50	56.50 ± 6.00	0.096	**0.031**
MET tot [ml/kg/min]	3270.00 ± 435.00	3502.50 ± 540.75	1.000	0.580
AHA Healthy Diet Score [au]	2.00 ± 1.00	2.00 ± 1.00	0.109	0.109
Perception of Stress [au]	5.00 ± 2.50	3.50 ± 2.50	**0.035**	**0.015**
4SQ score [au]	11.50 ± 5.50	8.00 ± 5.00	0.481	0.250
Perception of tiredness [au]	5.00 ± 2.00	3.50 ± 1.50	0.332	0.211
Perception of sleep quality [au]	7.00 ± 1.00	7.50 ± 0.50	0.549	0.261
Perception of overall health [au]	7.00 ± 1.00	8.00 ± 1.00	**0.021**	**0.021**
Perception of quality of life [au]	8.00 ± 1.00	8.00 ± 1.00	0.180	0.063

Abbreviations: WSR = Wilcoxon signed rank; SAP = systolic arterial pressure; DAP = diastolic arterial pressure; HR = heart rate; AHA = American Heart Association; 4SQ = subjective stress related somatic symptoms questionnaire; au = arbitrary unit. Significant values are evidenced in bold. Note: in the sign and WSR tests, the (paired) differences between values at T0 and T1 were computed for each variable X (i.e., $D=X_{1}-X_{0}$). Let θ be the median of variable D, regarded as the MRT effect. By those tests, the null hypothesis of zero MRT effect on the distribution of variable *D* is tested against the presence of MRT effect, i.e., $H_{0}:\theta =0$ vs. $H_{1}:\theta \neq 0$.

**Table 2:** Representative descriptive indices of RR interval variability indices at rest and their changes induced by active orthostatism: Summary statistics (median ± MAD at T0 and T1) and two-tailed non-parametric statistical tests.

Variables	Median ± MAD (Time 0)	Median ± MAD (Time 1)	Sign Test Significance	WSR Test Significance
HR mean [b/min]	64.50 ± 5.34	63.53 ± 5.52	0.815	0.442
RR [ms]	930.23 ± 72.12	945.60 ± 87.25	0.815	0.246
RRTP [msec^2^]	5983.85 ± 1950.13	5534.85 ± 2290.72	0.481	0.580
RR LFa [msec^2^]	1250.99 ± 577.54	1294.84 ± 671.00	0.481	0.229
RR LFnu [nu]	30.75 ± 6.92	30.10 ± 7.35	0.096	0.212
RR HFa [msec^2^)	2148.36 ± 1652.86	1865.16 ± 931.10	1.000	0.325
RR HFnu [nu]	61.66 ± 5.28	52.95 ± 10.43	0.481	0.468
RR LF/HF [.]	0.52 ± 0.17	0.58 ± 0.26	0.815	0.632
RR HFHz [Hz]	0.31 ± 0.04	0.33 ± 0.05	0.815	0.617
ΔHR mean [b/min]	14.99 ± 4.28	31.79 ± 8.25	**<0.001**	**<0.001**
ΔRR [ms]	−160.39 ± 72.95	−311.39 ± 64.87	**<0.001**	**<0.001**
ΔRRTP [msec^2^]	−2737.26 ± 2025.85	−3791.11 ± 1477.03	0.096	0.130
ΔRR LFa [msec^2^]	−46.28 ± 669.61	−295.09 ± 462.59	0.481	0.325
ΔRR LFnu [nu]	34.43 ± 10.35	55.96 ± 11.05	**0.031**	**0.014**
ΔRR HFa [msec^2^)	−1877.13 ± 930.20	−1671.91 ± 1046.77	0.481	0.832
ΔRR HFnu [nu]	−30.73 ± 12.07	−44.34 ± 10.90	0.815	0.468
ΔRR LF/HF [.]	2.50 ± 2.05	7.72 ± 6.40	0.096	**0.005**
ΔRR HFHz [Hz]	0.01 ± 0.01	−0.02 ± 0.06	0.481	0.375
ANSI	35.87 ± 13.99	57.55 ± 28.88	0.238	**0.030**

Abbreviations: WSR = Wilcoxon signed rank; HR mean = mean values of heart rate calculated considering continuous RR intervals recordings during rest; RR = RR interval; VAR_RR_ = RR interval variance; LF = low frequency component of RR variability; a = absolute value; HF = high frequency component of RR Variability; nu = normalized unit; LF/HF = LF_RR_ on HF_RR_ ratio; Δ = stand-rest difference; ANSI = autonomic nervous system index. Significant values are evidenced in bold. Note: the sign and WSR tests were used as described below in Table 1.

## Data Availability

Data will be available on justified request. We have not uploaded the data considering the paucity of patient numbers (elite athletes well known by the community) which may not guarantee privacy.
